# Supplementary material for: The interactome of intact mitochondria by cross-linking mass spectrometry provides evidence for coexisting respiratory supercomplexes
Source: Mol Cell Proteomics. 2017 Dec 8;17(2):216–32. doi: 10.1074/mcp.RA117.000470 (PMC5795388; doi:10.1074/mcp.RA117.000470)
Supplement: Supplemental Data [file supp_RA117.000470_133922_0_supp_23978_fzffwf.zip › spectra_annotation/mito_DR_spectra_annotation/8-1-7-1-10-1.pdf]

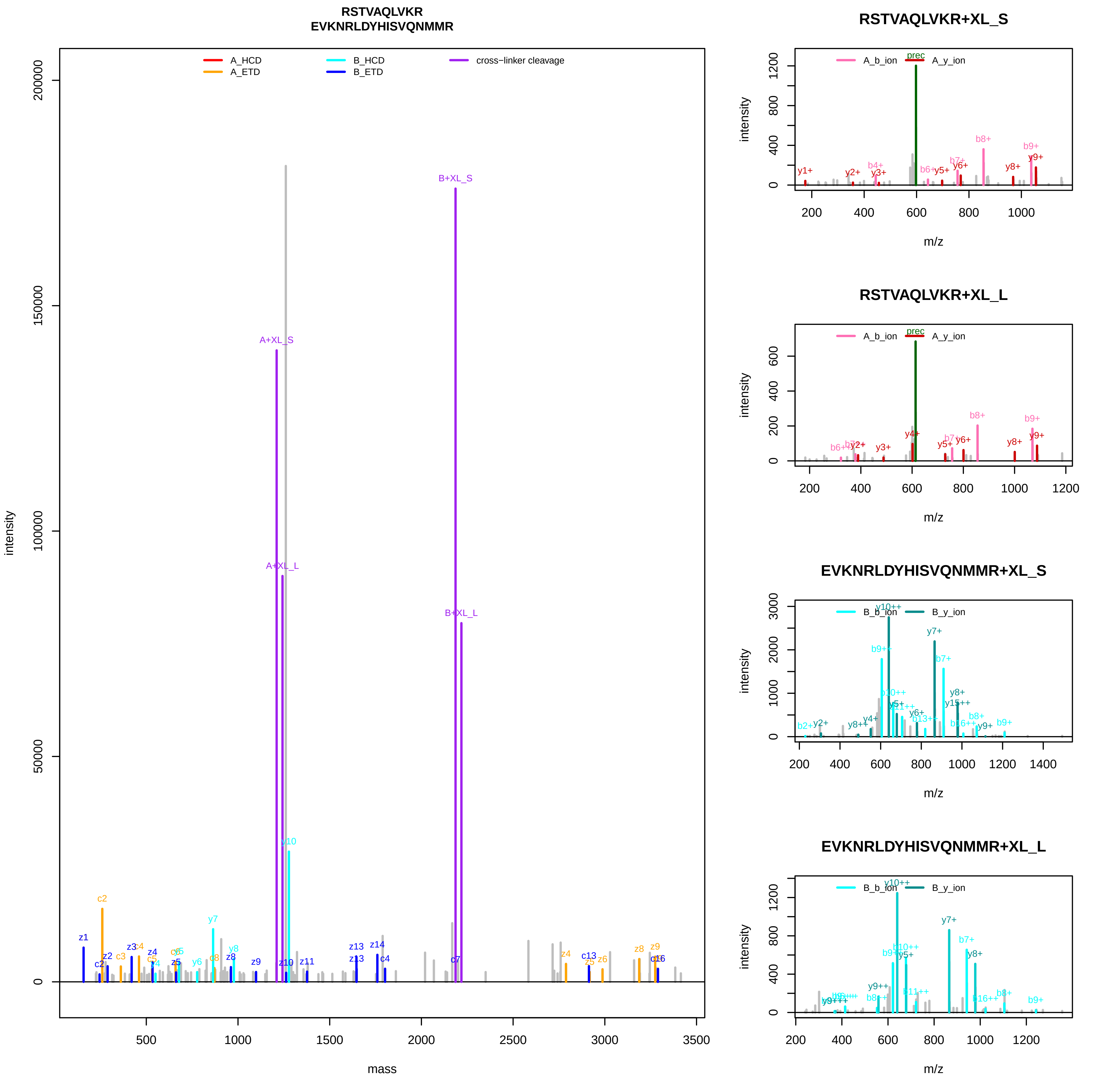

RSTVAQLVKR
EVKNRLDYHISVQNMMR
A_HCD
A_ETD
B_HCD
B_ETD
cross−linker cleavage
B+XL_S
A+XL_S
A+XL_L
B+XL_L
y10
y7
y8
c2
z1
z2
z3
c3
z4
z5
y6
c8
z8
z9
z10
z11
z13
z14
c4
c7
c13
z4
z5
z6
z8
z9
c16
intensity
mass
RSTVAQLVKR+XL_S
RSTVAQLVKR+XL_L
EVKNRLDYHISVQNMMR+XL_S
EVKNRLDYHISVQNMMR+XL_L
A_b_ion
A_y_ion
B_b_ion
B_y_ion
prec
y10++
y7+
b7+
b9++
y8+
b8+
b9+
intensity
m/z
